# Hyponatremia in Critically Ill Patients Due to Continuous Venovenous Hemofiltration With Diluted Sodium Citrate

**DOI:** 10.1097/MAT.0000000000002330

**Published:** 2024-10-22

**Authors:** Francesco Zadek, Beatrice Brunoni, Francesca Mulazzani, Francesco Minotti, Loredana Faraldi, Francesca Tardini, Riccardo Giudici, Stefania Paccagnini, Maria Luisa De Angelis, Roberto Fumagalli, Thomas Langer

**Affiliations:** From the * Department of Medicine and Surgery, University of Milan-Bicocca, Monza, Italy; † Department of Anesthesia and Intensive Care Medicine, Niguarda Ca’ Granda, Milan, Italy; ‡ SC Analisi Chimico Cliniche ASST Grande Ospedale Metropolitano Niguarda, Milan, Italy.

**Keywords:** continuous renal replacement therapy, sodium citrate, acute kidney injury, hyponatremia, intensive care unit

## Abstract

Continuous venovenous hemofiltration (CVVH) is frequently performed in critically ill patients using diluted citrate for regional anticoagulation. The impact of this renal replacement strategy on plasma sodium has not been evaluated yet. Our aim was therefore to assess the period prevalence of hyponatremia (sodium <135 mmol/L) during CVVH and discuss possible underlying mechanisms. After 48 hours of treatment, 70% of the 27 oligo-anuric critically ill patients were hyponatremic, despite the use of dialysis fluid bags (Regiocit 18/0, Phoxilium by Baxter, Deerfield, IL, and Multibic K2 by Fresenius Medical Care AG & Co. KGaA, Bad Homburg, Germany) with sodium content of 140 mmol/L. Indeed, sodium decreased from 142 ± 7 to 135 ± 3 mmol/L, *p* < 0.001. Sodium concentrations of employed dialysis bags were confirmed using ion chromatography. However, ionized sodium of Regiocit measured with a direct-ion selective electrode (ISE) resulted lower (~118 mmol/L), suggesting the presence of sodium-to-citrate complexes. Possible mechanisms explaining the hyponatremia development could therefore include: i) plasma water dilution; ii) a reduced Gibbs-Donnan effect, given the low albumin concentration (2.6 ± 0.8 g/dl) of our critically ill patients; iii) a negative sodium balance due to the loss of sodium-to-citrate complexes across the filter. The clinical implications of the described hyponatremia and the different contributions of the hypothesized mechanisms need to be addressed in future studies.

## Graphical abstract

**Figure:**
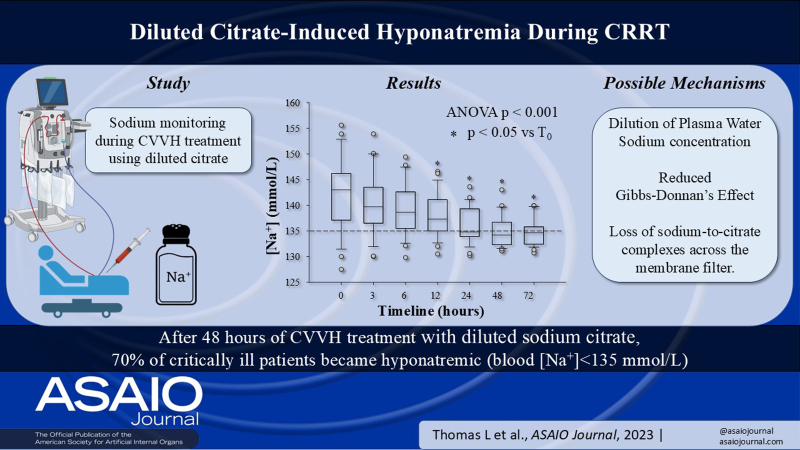

Continuous renal replacement therapy (CRRT) is an extracorporeal treatment employed in approximately 10–15% of patients admitted to the intensive care unit (ICU),^1,2^ mainly for the treatment of acute kidney injury and conditions leading to severe acid-base or electrolyte disorders.^3^

Continuous venovenous hemofiltration (CVVH) is a CRRT modality exploiting convection, *i.e.*, the removal of solutes together with water (ultrafiltrate).^4^ Therefore, CVVH implies administering a significant amount of fluid to the patient through the extracorporeal circuit to replace the removed water and electrolytes. These fluids are infused both as reinfusion fluids and regional citrate anticoagulation (RCA). Consequently, employed CVVH fluid bags markedly influence plasma electrolytes.

The development of electrolyte derangements in critically ill patients treated with CRRT has been studied by several authors, focusing on hypophosphatemia, hypo-/hyperkalemia, and hypomagnesemia.^5–9^ So far, less attention has been dedicated to sodium alterations. Of note, the administration of sodium is frequently linked to that of citrate, a well-established technique to perform regional anticoagulation and avoid complications associated with systemic heparin infusion. The use of concentrated sodium citrate solutions (citrate 136 mmol/L, sodium 420 mmol/L) has been associated with the development of hypernatremia.^10–13^ A more diluted citrate solution was introduced in 2001 to address this issue.^14^ This solution contains 18 mmol/L of citrate bound to 54 mmol/L of sodium, to which sodium chloride is added to reach a sodium concentration of 140 mmol/L. A direct consequence of the use of diluted citrate is that the amount of fluid administered to patients for RCA is markedly higher than the concentrated form. Consequently, this fluid acts both as an anticoagulant and as a replacement fluid administered in predilution.^14^

In our clinical practice, we observed that some patients treated with CVVH using diluted citrate developed hyponatremia. We therefore designed the current study with the primary aim of assessing the period prevalence of hyponatremia during CVVH treatment using diluted citrate. The secondary aim was to investigate the underlying mechanisms of hyponatremia development, analyzing the sodium variations along the CRRT circuit and the sodium content of employed fluid bags.

## Materials and Methods

To address the period prevalence of hyponatremia during CVVH treatment using diluted citrate, we conducted a single-center, prospective, observational study in three ICUs of the Niguarda Hospital in Milan, Italy. The institutional review board approved the study (CEMIA3, # 16022022, December 15, 2021). Informed consent was obtained according to Italian regulations.

The study included oligo-anuric adult patients (urinary output <0.5 ml/kg/h for 12 hours) with acute kidney injury admitted to the ICU and who required CVVH treatment.

Exclusion criteria were the following: the development of hemodynamic instability requiring the infusion of large amounts of crystalloids (>3 L/day), exogenous sodium correction, and CVVH treatment duration <48 hours (a posteriori exclusion criteria).

Demographic information, relevant medical history, and clinical parameters (cause of ICU admission, sequential organ failure assessment [SOFA], presence of mechanical ventilation, vasopressor administration, vasoactive inotropic score [VIS], and diuresis in the last 12 hours) were recorded at study inclusion. Moreover, data regarding (intravenous and enteral) fluid administration and fluid balance were collected daily.

Continuous venovenous hemofiltration was performed using the PrisMax equipped with AN69 ST150 hollow fiber filter (Baxter International Inc, Baxter Healthcare SA, Zurich, Switzerland). Diluted citrate (Regiocit, trisodium citrate 18/0, Baxter Healthcare Corporation, Deerfield, IL) was administered for RCA. Phoxilium (Baxter International Inc) or Multibic K2 (Fresenius Medical Care AG & Co. KGaA) were administered as replacement solutions in postdilution modality and were chosen according to clinical judgment. The electrolyte compositions of the employed CRRT fluid bags are reported in Table S1, Supplemental Digital Content, http://links.lww.com/ASAIO/B347. Notably, all employed solutions have a reported sodium concentration of 140 mmol/L.

Data regarding CRRT settings were collected at baseline, 3, 6, 12, 24, 48, and 72 hours post-CVVH start (*T*_0_, *T*_3_, *T*_6_, *T*_12_, *T*_24_, *T*_48_, and *T*_72,_ respectively). At each time point, the following parameters were recorded: blood flow (ml/min), convective dose (ml/kg/h), citrate flow (ml/h), replacement fluid flow (ml/h), weight loss (ml/h), and cumulative effluent volume (ml).

Venous blood samples were collected at each time point from the dialysis catheter before the citrate infusion and analyzed using a blood gas machine (RapidPoint 500e; Siemens Healthineers, Milan, Italy). Additionally, at baseline, *T*_24_, *T*_48,_ and *T*_72,_ blood samples were sent to the central laboratory to measure creatinine, urea, phosphate, albumin, and osmolality (MIR-300-P; Emanuele Mires, Milan, Italy).

**Figure 1.**
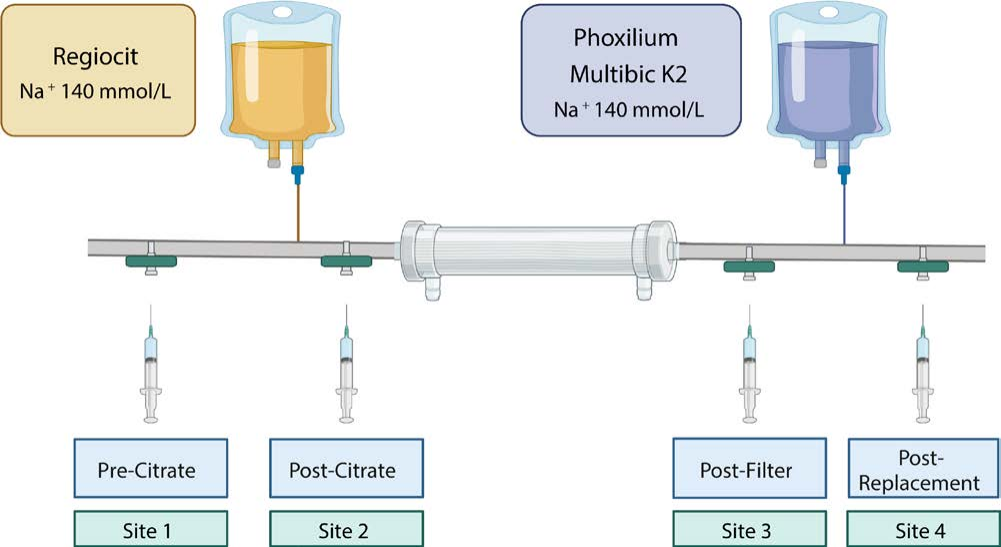
Graphical representation of CVVH circuit and site of blood sampling (site 1, site 2, site 3, and site 4). CVVH, continuous venovenous hemofiltration; Na^+^, sodium concentration. Created with BioRender.com.

Five minutes after the start of CVVH, intra-CRRT samples were collected. Sites for blood sampling access were coded according to the position along the circuit as follows: pre-citrate infusion (site 1), post-citrate infusion, pre-filter (site 2), post-filter (site 3), post-replacement fluid infusion (site 4) (Figure 1). A blood gas analysis was performed from each site, and osmolality was measured.

### Sodium Measurement Using Ion Chromatography and Estimation of Sodium-Citrate Complexes in Employed Fluid Bags

Ion chromatography analysis was used to measure sodium concentration in Regiocit and Phoxilium. Chromatographic separation was performed using a Metrohm Eco Ion Chromatograph (IC) system (Metrohm AG, Herisau, CH), equipped with a conductivity detector, a Metrohm Suppressor Module (MSM) conductivity suppressor and a Metrohm 863 Compact Autosampler. The IC was equipped with a Metrohm Metrosep A Supp 5 (4 mm × 250 mm) and a Metrosep A Supp 1 Guard/4.6 guard-column for anion separation and with a Metrosep C 6 (4 mm × 250 mm). The mobile phase was a 4 mmol/L nitric acid + 0.7 mmol/L oxalic acid aqueous solution, at a flow rate of 0. 9 ml/min, at room temperature (25°C). The injection volume was 10 µl. The eluent conductivity background was suppressed by using a Metrohm MSM cation-exchange membrane set in-line after the anionic column (no suppression was used for cations detection). It was regenerated with 100 mmol/L of sulfuric acid flowing countercurrently at a flow rate of 1. 5 ml/min.

Additionally, sodium concentration of Regiocit and Phoxilium was measured using a direct-ISE (RapidPoint 500e; Siemens Healthineers). The presence of sodium-citrate complex was estimated as the difference between sodium measured by ion chromatography (total sodium) and using direct-ISE (ionized sodium). In addition, osmolality of the fluid bags was measured (MIR-300-P; Emanuele Mires) and compared with theoretical osmolality.

### Sample Size

The primary outcome of the study was the period prevalence of hyponatremia at 48 hours from the start of CVVH, hypothesizing a significant difference compared with the prevalence (15%) of hyponatremia of our own ICU population (unpublished data). Notably, this percentage is in line with the prevalence reported in the literature.^15^ We calculated that a sample size of 25 would allow the identification of a 35% absolute difference in hyponatremia period prevalence (50% *vs.* 15%) using a binomial proportion test (*β* = 0.95, *α* = 0.05). Given the presence of a posteriori exclusion criteria, we increased the sample size to 30.

### Statistical Analysis

Data are expressed as mean ± standard deviation, median [interquartile range], or frequency (percentage), unless otherwise stated. The normality of the population was tested using the Shapiro-Wilk test. Comparison between two continuous variables was performed *via* paired *t* test or signed rank sum test, as appropriate. Comparisons between multiple groups over time were performed using one-way or two-way analysis of variance (ANOVA) using Bonferroni’s correction or Friedman ANOVA on ranks for repeated measures using Dunn’s correction. The relationship between continuous variables was investigated using linear regression and Pearson’s correlation.

Analyses were performed on both the overall population and after dividing the population into three subgroups, according to baseline sodium concentration, *i.e*., hyponatremia (sodium <135 mmol/L), normonatremia (sodium 135–145 mmol/L), and hypernatremia (sodium >145 mmol/L).

Statistical significance was defined as *p* < 0.05. Analyses were performed using Stata statistical software (Stata Statistical Software 16.0; StataCorp, College Station, TX). Graphs were drawn with SigmaPlot 11.0 (Systat Software, San Jose, CA) and images with Biorender (Toronto, Canada). The Strengthening the Reporting of Observational Studies in Epidemiology checklist was used.

## Results

The study enrolled 30 consecutive patients from January to November 2022. Twenty-seven patients (with an age range from 27 to 76 years, and 22% being female) were included in the analysis. Three patients were excluded due to discontinuation of CRRT before 48 hours.

A summary of the demographic characteristics and admission diagnoses is presented in Table 1.

**Table 1.** Demographics and Cause of ICU Admission of the Study Patients

Variables	Population (n = 27)
Age (years)	62 [27, 76]
Female, n (%)	6 (22)
Body mass (kg)	89 ± 15
Height (m)	1.7 ± 0.8
BMI (kg/m^2^)	29.0 ± 5.4
SOFA score	10 ± 3
Mechanical ventilation, n (%)	20 (77)
Vasopressor administration, n (%)	16 (59)
VIS	19 ± 16
Cause of ICU admission	
Septic shock, n (%)	9 (33)
Burns, n (%)	4 (15)
ARDS, n (%)	6 (22)
Trauma, n (%)	3 (11)
MALA, n (%)	2 (7)
Hemorrhagic shock, n (%)	1 (4)
Postoperative acute kidney injury, n (%)	1 (4)

Data are expressed as mean ± standard deviation, median [range], frequency (percentage).

ARDS, acute respiratory distress syndrome; BMI, body mass index; ICU, intensive care unit; MALA, metformin-associated lactic acidosis; SOFA, sequential organ failure assessment; VIS, vasoactive inotropic score.

Baseline serum creatinine concentration was 4.2 ± 2.7 mg/dl, with a urea of 163 ± 68 mg/dl and a residual diuresis of 0.2 ± 0.2 ml/kg/h over the last 12 hours before CRRT initiation. The initial convective dose was 31 ± 5 ml/kg/h, with a blood flow of 144 ± 13 ml/min, a citrate flow of 1,441 ± 125 ml/h, and a replacement solution flow of 1,424 ± 171 ml/h. Throughout the treatment, the CVVH settings remained constant (Table 2). Patients remained oligo-anuric during the 72 hours of CVVH treatment. Daily fluid balance and free water administration are reported in Table 3. Details regarding administered fluids and their osmolarity are reported in Table S2, Supplemental Digital Content, http://links.lww.com/ASAIO/B347.

**Table 2.** CVVH Settings During the First 48 Hours of Treatment

Variables	Baseline	48 Hours
Convective dose (ml/kg/h)	31 ± 5	32 ± 5
Blood flow (ml/min)	144 ± 12	143 ± 10.9
Weight loss (ml/h)	31 ± 38	71 ± 58
Citrate		
Citrate flow (ml/h)	1,441 ± 125	1,423 ± 111
Volume infused (ml)		66,248 ± 12,967
Replacement fluid		
Phoxilium, n(%)	7 (26)	
Multibic K2, n(%)	20 (74)	
Replacement fluid flow (ml/h)	1,424 ± 171	1,446 ± 105
Volume infused (ml)		70,874 ± 4,334
Calcium replacement		
CaCl 10% (ml/h)	4.0 ± 0.5	4.1 ± 0.8

Data are expressed as a mean ± standard deviation, frequency (percentage).

CaCl, calcium chloride; CVVH, continuous venovenous hemofiltration.

**Table 3.** Trends of the Main Plasma Electrolytes and Acid-Base Parameters During the First 72 Hours of CVVH Treatment

Variables	Baseline	24 Hours	48 Hours	72 Hours	*p* Value
Na^+^ (mmol/L)	142 ± 7	136 ± 3	135 ± 3	135 ± 2	<0.001
K^+^ (mmol/L)	4.4 ± 0.7	4.0 ± 0.3	4.1 ± 0.3	4.0 ± 0.3	0.053
Cl^−^ (mmol/L)	104 ± 6	99 ± 2	98 ± 2	98 ± 2	<0.001
Osmolality (mOsm/kg)	328 ± 22	300 ± 12	295 ± 10	291 ± 7	<0.001
Glucose (mg/dl)	142 ± 51	131 ± 30	133 ± 30	132 ± 28	0.38
Urea (mg/dl)	158 ± 73	83 ± 43	60 ± 32	51 ± 30	<0.001
Lactate (mmol/L)	2.9 ± 4.2	1.4 ± 0.7	1.3 ± 0.7	1.2 ± 0.6	0.009
Phosphate (mg/dl)	5.3 ± 3.1	3.7 ± 1.2	3.0 ± 1.2	2.9 ± 1.1	<0.001
Creatinine (mg/dl)	4.2 ± 2.7	2.4 ± 1.8	1.5 ± 0.7	1.7 ± 0.0	<0.001
Albumin (g/dl)	2.7 ± 0.6	2.9 ± 0.5	2.8 ± 0.4	2.6 ± 0.7	0.14
pH	7.30 ± 0.12	7.38 ± 0.06	7.39 ± 0.06	7.4 ± 0.06	0.001
HCO_3_^−^ (mmol/L)	23 ± 5	28 ± 3	29 ± 3	29 ± 2	<0.001
Base excess (mmol/L)	−3.2 ± 6.7	3.2 ± 3.1	4.2 ± 2.4	5.1 ± 4.4	<0.001
Urinary output (ml/kg/h)	-	0.1 [0.0–0.1]	0.1 [0.0–0.3]	0.1 [0.0–0.3]	0.18
Fluid balance (ml)	-	−115 [−1,027 to 561]	68 [−705 to 920]	−106 [−912 to 910]	0.94
Intravenous fluid input (ml)	-	1,071 [843–1,610]	1,090 [745–1,483]	1,117 [747–1,553]	0.78
Intravenous fluid [Na^+^] (mmol/L)	-	138 [113–144]	136 [120–144]	141 [126–145]	0.84
Day free water (ml)	-	391 [171–894]	686 [289–863]	509 [181–743]	0.89

Data are expressed as mean ± standard deviation or median [interquartile range]. The ANOVA *p* values for repeated measures between *T*_0_, *T*_24_, *T*_48_, and *T*_72_ were calculated for each variable.

Cl^−^, chloride concentration; CVVH, continuous venovenous hemofiltration; HCO_3_^−^, bicarbonate concentration; K^+^, potassium concentration; Na^+^, sodium concentration.

### Sodium Variations During Continuous Venovenous Hemofiltration

**Figure 2.**
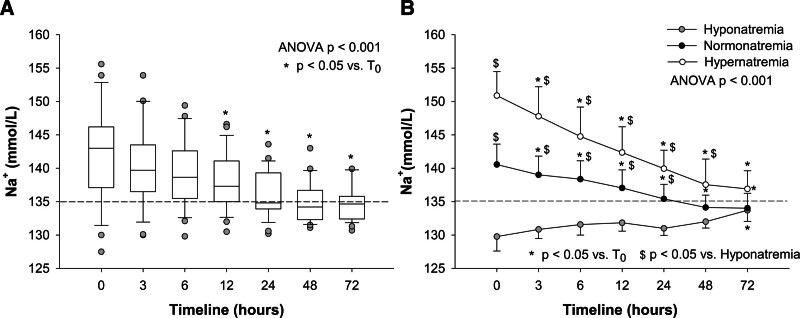
Box plot graph of plasma sodium variations during the first 72 hours from CVVH start (**A**). Notably, the different time points were not represented on the x-axis using a linear scale. *p* Value of one-way ANOVA for repeated measures <0.001. *p < 0.05 *vs.* baseline (*T*_0_). Trends of plasma sodium concentration of the population divided according to baseline sodium concentration: hypernatremia (>145 mmol/L, white dots), normonatremia (between 135 and 145 mmol/L, black dots), and hyponatremia (<135 mmol/L, gray dots) (**B**). *p* Value of two-way ANOVA for repeated measures <0.001. *p < 0.05 *vs.* baseline (*T*_0_); ^$^*p* < 0.05 *vs.* hyponatremia. ANOVA, analysis of variance; CVVH, continuous venovenous hemofiltration; Na^+^, sodium concentration.

At treatment initiation, hypernatremia was observed in seven (26%), normonatremia in 17 (63%), while three patients (11%) were hyponatremic (151 ± 4, 141 ± 3, and 130 ± 2 mmol/L of sodium, respectively). Overall, baseline sodium concentration was 142 ± 7 mmol/L and decreased to 135 ± 3 mmol/L (*p* < 0.001) after 48 hours of CVVH treatment. The period prevalence of hyponatremia at 48 hours was 70% (19/27), *i.e.*, significantly higher (*p* = 0.02) than the 15% prevalence of hyponatremia in our ward. Considering the three hyponatremic patients, the incidence of hyponatremia was 66% (16/24 patients). The time course of sodium variations in the study population is presented in Figure 2A. Among patients experiencing hyponatremia, the mean plasma sodium concentration was 133 ± 1 mmol/L at 48 hours.

Importantly, distinct trends in plasma sodium variations were noted according to baseline sodium concentration (Figure 2B). In particular, within the first 24 hours of CVVH treatment, a significative (*p* < 0.001) sodium reduction of 11 ± 3 mmol/L was observed in hypernatremic patients. During the same timeframe, normonatremic patients exhibited a reduction of sodium concentrations of 5 ± 3 mmol/L (*p* = 0.002). Conversely, no significant sodium variations were observed in hyponatremic patients after 48 hours of treatment. In addition, the incidence of hyponatremia differed among the three subgroups after 48 hours: hyponatremia developed only in 30% of the hypernatremic patients, while it occurred in 87% of normonatremic patients.

**Figure 3.**
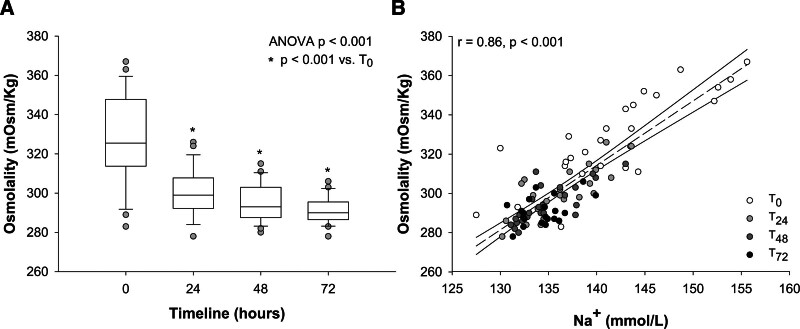
Box plot graph of plasma osmolality variations during the first 72 hours from CVVH start (**A**). *p* value of one-way ANOVA for repeated measures <0.001. *p < 0.05 *vs.* baseline (*T*_0_). Linear regression between sodium and osmolality of study samples (**B**). Samples were divided for timepoint *T*_0_ (white dots), *T*_24_ (light gray dots), *T*_48_ (gray dots), and *T*_72_ (black dots). Pearson’s *r* of 0.86, *p* < 0.001. ANOVA, analysis of variance; CVVH, continuous venovenous hemofiltration; Na^+^, sodium concentration.

Within the first 48 hours, the overall reduction in sodium concentration was paralleled by a decrease in plasma osmolality from 328 ± 23 to 295 ± 10 mOsm/kg (*p* < 0.001) (Figure 3A). Of note, baseline values present a wide range of both sodium and osmolality, while these parameters tend to converge during the treatment (Figure 3B). Partitioning osmolality in its major components, the observed variation was equally justified by a reduction in sodium and urea (see Table S3, Supplemental Digital Content, http://links.lww.com/ASAIO/B347).

### Variation of Acid-Base Parameters and Other Analytes

Blood acid-base parameters and analytes at *T*_0_, *T*_24_, *T*_48_, and *T*_72_ are reported in Table 3.

On average, at baseline, patients were characterized by metabolic acidosis, which was corrected within the first 24 hours of treatment (pH from 7.30 ± 0.12 to 7.38 ± 0.06 and standard base excess (BE) from −3.2 ± 6.7 to 3.2 ± 3.1 mmol/L). At *T*_48_ and *T*_72_, these parameters remained stable.

### Sodium and Osmolality Changes Within the Continuous Renal Replacement Therapy Machine

To investigate the mechanisms underlying the development of hyponatremia, we analyzed sodium and osmolality changes occurring along the CRRT circuit.

**Figure 4.**
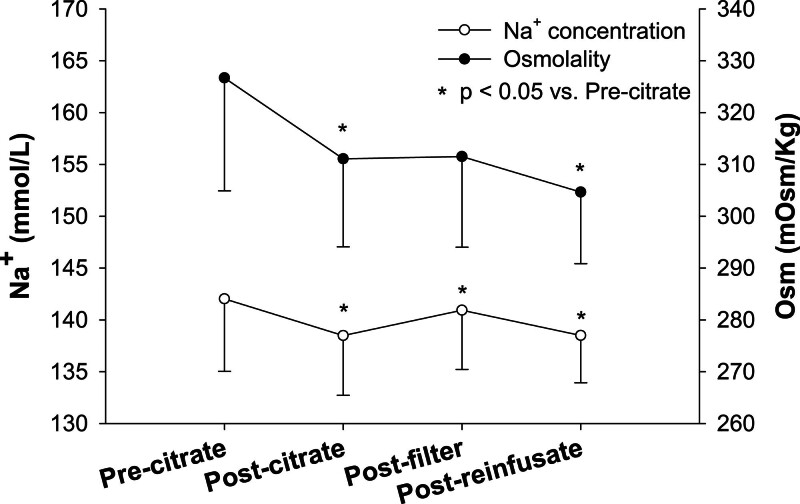
Sodium concentration (white dots) and osmolality (black dots) were measured within the circuit 5 minutes after CVVH started. The blood samples were collected pre-citrate, post-citrate, post-filter, and post-replacement solution. Data are represented as a mean and standard deviation. *p* value of one-way ANOVA for repeated measures <0.001. *p < 0.05 *vs.* pre-citrate. ANOVA, analysis of variance; CVVH, continuous venovenous hemofiltration; Na^+^, sodium concentration.

Two main sodium concentration reductions were observed within the circuit (Figure 4). The first occurred between site 1 and site 2 (142 ± 7 *vs*. 138 ± 6 mmol/L, *p* < 0.001), *i.e.*, after the addition of diluted citrate. The second sodium drop was observed between site 3 and site 4 (141 ± 6 *vs.* 138 ± 4 mmol/L, *p* < 0.001), *i.e.*, after the addition of replacement fluid.

Similarly, a reduction in osmolarity was observed between sites 1 and 2 (328 ± 22 *vs.* 311 ± 17 mOsm/kg, *p* < 0.001) and sites 3 and 4 (312 ± 17 *vs.* 305 ± 14 mOsm/kg, *p* < 0.001). Of note, sodium increased between site 2 and site 3 (138 ± 6 mmol/L *vs.* 141 ± 6 mmol/L, *p* < 0.001).

Sodium measured through ion chromatography and direct-ISE was 137.3 ± 1.1 and 117.8 ± 0.2 mmol/L for Regiocit, while it was 137.3 ± 1.0 and 133 ± 2 mmol/L for Phoxillium. Declared and measured osmolality were 244 and 216 ± 1 mOsm/kg for Regiocit, and 294 and 290 ± 3 mOsm/kg for Phoxillium.

## Discussion

The major finding of our study is a very high period prevalence of hyponatremia in critically ill patients undergoing CVVH using regional anticoagulation with diluted citrate. Considering the declared sodium concentration of all used dialysis fluid bags, a trend toward 140 mmol/L of sodium could have been expected. However, recorded sodium concentrations consistently pointed towards lower values, frequently below 135 mmol/L. Of note, this sodium drop was associated with a consensual reduction in osmolality, which, however, rarely reached values below 280 mOsm/kg.

Is this hyponatremia true? In line with the most recent guidelines on the diagnosis of hyponatremia, we used a point-of-care blood gas analyzer employing direct ISE for the measurement of sodium.^16^ This methodology analyzes whole blood and, unlike indirect ISE (typical of central laboratories), does not require predilution of the sample.^17^ The straightforward consequence is a markedly reduced impact of potential confounding factors, such as hypoalbuminemia or hypertriglyceridemia, leading to pseudohyper- or hyponatremia.^18,19^

Besides the measurement of plasma sodium, the algorithms clinically employed to identify the cause of hyponatremia require the measurement of osmolality to exclude the presence of other osmoles, such as glucose and mannitol.^20,21^ Of note, these conditions should be easily identified with basic laboratory analyses and clinical history. The most important part of the diagnostic algorithm, however, relies on the analysis of urine (sodium and osmolality), as the kidney is usually directly or indirectly responsible for the development of hyponatremia. The role of the kidneys in our patients is negligible, as they were oligoanuric at baseline, and their kidney function did not recover within the first 48–72 hours. In addition, it is important to underline that the abovementioned algorithms cannot be applied to anuric patients. Furthermore, by excluding patients who received high amounts of fluids and exogenous sodium corrections, we limited the role of possible external confounding factors. Notably, the daily administration of free water was only around 500 ml. In summary, given these premises, the timing of hyponatremia development, and considering the high volume (around 70 L) of water and electrolytes exchanged daily through the CRRT machine, it is conceivable to attribute the main role of the development of hyponatremia to the blood purification system. The underlying mechanisms, however, remain to be defined. Indeed, while evolution has equipped the kidneys with sophisticated systems to uncouple the excretion of water and electrolytes,^22–24^ this is not the case for the artificial filter, where ultrafiltration is driven solely by hydrostatic pressure. Let us, therefore, analyze the potential sources of water gain or sodium loss within the CRRT system.

The first mechanism, already described mainly in the context of intermittent dialysis, is the dilution of plasma water.^25^ Indeed, normal plasma is composed of approximately 93% of water and 7% of nonaqueous components (*i.e.*, proteins and lipids). The sodium concentration measured by analyzers represents the average value per liter of solution, which implies that its value in plasma water is higher. For example, assuming a normal protein and lipid concentration, a plasma sample with 140 mmol/L of sodium would have, in theory, a plasma water concentration of sodium of 150.5 mmol/L (140/0.93). With its treatment, CRRT only affects the watery part of plasma, substituting it with a solution having lower sodium concentrations and, therefore, most likely contributing to the development of plasma hyponatremia. However, critically ill patients have typically a reduced nonaqueous component, as demonstrated by hypoalbuminemia.^26^ This condition, *per se*, blunts the plasma water dilution effect. A second relevant mechanism acting on sodium concentration is the Gibbs-Donnan effect. Indeed, the presence of negatively charged molecules, such as albumin, which are unable to cross the filter, generates an uneven electrolyte distribution between the blood and ultrafiltrate compartments of the CRRT filter. This fact favors the retention of positively charged ions, such as sodium, in the blood.^27^ Our clinical data show an increase in sodium concentration along the filter (Figure 4), corroborating the presence of the Gibbs-Donnan effect. However, given the low albumin concentration of our critically ill patients, it is conceivable that the net sodium-retaining effect is reduced as compared with patients with normal protein concentration, contributing to the observed hyponatremia. Finally, while an increased loss of ionized sodium through ultrafiltrate fluid can be excluded due to the presence of the Gibbs-Donnan effect, sodium could be lost bound to citrate. Sodium of the employed fluid bags was measured both with direct-ISE and ion chromatography. We found a sodium concentration of 118 mmol/L with direct-ISE a value markedly lower than the one obtained with ion chromatography. These results, *i.e.*, approximately 20 mmol/L discrepancy in sodium concentration found in Regiocit between the two measurements, support the presence of a relevant amount of sodium-to-citrate binding, possibly due to the different dissociation constants of citrate.^28^ In addition, the measured osmolality of Regiocit, which is lower than the declared value, further points toward a reduced presence of ionized sodium. Of note, differences in measured and declared sodium and osmolality of Phoxilium *i.e.*, a dialysis fluid that does not contain citrate, were modest.

These data taken together suggest that the addition of Regiocit actually provides 140 mmol/L of sodium, which is however in two different forms: approximately 85% of the sodium seems to be in ionized form, while the remaining 15% is most likely bound to citrate. This latter form of sodium undergoes ultrafiltration and could contribute to a neglected sodium loss, possibly leading to a negative sodium balance. Interestingly, this sodium loss is not detectable by using ISE. In conclusion, a combination of the mechanisms described above is reasonably responsible for the observed hyponatremia.

### Clinical Implications

We have described a novel, unexpected cause of hyponatremia during CVVH treatment despite the exclusive use of dialysis fluid bags containing 140 mmol/L of sodium. To our knowledge, while other studies report a sodium reduction during CRRT treatment with diluted citrate,^29–32^ this is the first study systematically addressing this topic. Our study suggests that the hyponatremia induced by CVVH could be frequent and that this condition should be considered in the differential diagnosis of hyponatremia in the intensive care setting. The association between hyponatremia and mortality in critically ill patients is well-known.^33^ Our data are, however, insufficient to evaluate the clinical consequences of the observed electrolyte derangement. As this electrolyte disorder is not an expression of an underlying pathologic process, but a consequence of medical treatment, we doubt that it might overall impact patients’ outcomes. However, our results should be considered when dealing with patients with hypernatremia or increased intracranial pressure. Indeed, in both cases, a rapid decrease in sodium concentration could potentially increase the risk of developing brain edema.^34^ Nevertheless, adding sodium chloride to the fluid bags used for CVVH is an easy and effective strategy to avoid hyponatremia.^35^

A final consideration concerns the sodium-rich environment of the ICU.^36^ Indeed, ICU patients usually receive extremely high quantities of sodium per day due to abundant intravenous isotonic fluids, drug administration, and fluid creep, potentially leading to sodium and fluid overload.^23,37–39^ The described CVVH system could provide an efficient way to remove sodium extracorporeally.

### Limitations

Some limitations of our study need to be mentioned. First, it is a mono-centric study performed on a relatively small number of patients. Second, we have studied only one modality of CRRT, namely CVVH. We can therefore draw no conclusions on the development of hyponatremia using continuous venovenous hemodialysis (CVVHD) and hemodiafiltration (CVVHDF). Third, the infusion of more than 3 L per day of crystalloid is not rare, particularly in the acute phase of the disease. We excluded patients receiving high amounts of fluids to avoid confounding factors. It is conceivable that the incidence of hyponatremia would be reduced in case of abundant fluid resuscitation with isotonic crystalloids. Finally, and most importantly, our study did not address the clinical impact of the described hyponatremia, and it does not provide definitive proof of the underlying mechanisms. In particular, the clearance of sodium-to-citrate complexes needs to be investigated in detail in further studies, addressing also the role of calcium in this context.

## Conclusions

We have described a high period prevalence of hyponatremia in critically ill patients undergoing CVVH with diluted sodium citrate, which should be taken into consideration in this specific patient population. While the impact on patients’ outcomes of this novel entity has yet to be defined, it seems reasonable to avoid it in patients with brain edema and to slow down its development in patients with hypernatremia.

## Acknowledgments

The authors are indebted to all the residents, nurses, and physicians of the general intensive care units of Niguarda Hospital for their valuable support; Prof. Norberto Manfredi and Dr. Chiara Boldrini of the Department of Materials Science of the University of Milano-Bicocca, Milan, Italy, for the performance of ion-chromatography measurements.

## Supplementary Material
