# Supplementary material for: Hyponatremia in Critically Ill Patients Due to Continuous Venovenous Hemofiltration With Diluted Sodium Citrate
Source: ASAIO J. 2024 Oct 22;71(4):355–62. doi: 10.1097/MAT.0000000000002330 (PMC11949219; doi:10.1097/MAT.0000000000002330)
Supplement: Supplementary file 1 [file mat-71-355-s001.pdf]

Hyponatremia in critically ill patients due to continuous venovenous hemofiltration with diluted sodium citrate

Supplementary Materials

Table S1. Composition and theoretical osmolarity of Regiocit 18/0, Multibic K2, and Phoxilium, as reported by the manufacturer.

Variables	Multibic K2 (Fresenius)	Phoxilium (Baxter)	Regiocit 18/0 (Baxter)
**Na^+ (mmol/L)**	140	140	140
**Cl^- (mmol/L)**	111	115.9	86
**K^+ (mmol/L)**	2	4	0
**Ca^{2+} (mmol/L)**	1.5	1.24	0
**Mg^{2+} (mmol/L)**	0.5	0.75	0
**$H_2PO_4^-$ (mmol/L)**	0	1	0
**HCO_3^- (mmol/L)**	35	32	0
**$C_6H_5O_7^{3-}$(mmol/L)**	0	0	18
**Glucose (mg/dL)**	100	0	0
**Osmolarity (mOsm/L)**	296	294	244

Ca^{2+}= calcium concentration; Cl^-= chloride concentration; HCO_3^- = bicarbonate concentration; $H_2PO_4^-$= phosphate concentration; K^+= potassium concentration; Mg^{2+}= magnesium concentration; Na^+= sodium concentration; $C_6H_5O_7^{3-}$ = citrate concentration.

Table S2. Daily volume and type of intravenous fluids administered during the study period.

	NaCl 0.9%	Rehydrating III	Ringer Lactate	5% Dextrose
Na^+, mmol/L	154	140	130	0
Osmolarity, mOsm/L	308	312	278	278
Patients, n (%)	26 (96%)	19 (70%)	13 (48%)	23 (85%)
Daily volume infused, ml	532 [324; 794]	500 [400; 988]	435 [240; 966]	120 [72; 250]

Sodium and osmolarity are reported as declared by the manufacturer of each crystalloid. Na^+ = sodium concentration.

Table S3. Partitioning of plasma osmolarity on each day of treatment. Osmolality was measured by Freezing point, and Osmolar Gap was calculated between the difference of measured osmolality and calculated osmolarity.

	**Baseline**	**Day 1**	**Day 2**	**Day 3**	**p-value**
**Osmolality, mOsm/Kg**	328 ± 22	300 ± 12	295 ± 10	291 ± 7	<0.001
**2 x Na^+, mOsm/L**	284 ± 14	272 ± 7	270 ± 6	270 ± 5	<0.001
**Glucose, mOsm/L**	8 ± 3	7 ± 2	7 ± 2	7 ± 2	0.34
**Urea, mOsm/L**	26 ±12	13 ± 7	10 ± 5	8 ± 5	<0.001
**Osmolar Gap, mOsm/L**	10 ± 2	7 ± 2	8 ± 1	6 ± 1	0.48

Osmolality = measured osmolality (freezing point); 2 x Na^+= osmolarity deriving from electrolytes, calculated as twice the measured concentration of sodium; Glucose = osmolarity deriving from glucose; Urea = osmolarity deriving from urea; Osmolar Gap = difference between measured and calculated osmolarity.
